# *Fusarium graminearum* in Wheat—Management Strategies in Central Europe

**DOI:** 10.3390/pathogens14030265

**Published:** 2025-03-08

**Authors:** Weronika Giedrojć, Wioletta E. Pluskota, Urszula Wachowska

**Affiliations:** 1 Department of Entomology, Phytopathology and Molecular Diagnostics, Faculty of Agriculture and Forestry, University of Warmia and Mazury in Olsztyn, ul. Prawocheńskiego 17, 10-722 Olsztyn, Poland; urszula.wachowska@uwm.edu.pl; 2 Department of Plant Physiology, Genetics and Biotechnology, Faculty of Biology and Biotechnology, University of Warmia and Mazury in Olsztyn, ul. Michała Oczapowskiego 1A, 10-719 Olsztyn, Poland; wioletta.pluskota@uwm.edu.pl

**Keywords:** biopreparations, fungicides, Fusarium head blight, mycotoxins, wheat

## Abstract

The main aim of this study was to discuss and compare the threats associated with *F. graminearum* in wheat production in Poland and in other Central European countries. Wheat is one of the most widely cultivated crops in the world, and pathogens causing Fusarium head blight (FHB) pose the greatest threat to wheat production. Our knowledge of FHB has to be regularly expanded in order to explore the impacts of climate change, new wheat cultivars, and new fungicides on the prevalence of this disease. The pathogen’s resistance to fungicides was analyzed in a global context due to the relative scarcity of studies examining this problem in Central Europe (excluding Germany). This is an interesting research perspective because, despite a relatively large number of Polish studies on FHB, *F. graminearum* genotypes and the pathogen’s resistance to fungicides remain insufficiently investigated. The hemibiotrophic pathogen *Fusarium graminearum* causes particularly high losses in wheat cultivation due to its ability to produce mycotoxins that are dangerous to human health (mainly deoxynivalenol, DON), colonize plant residues in soil in the saprotrophic phase, and produce spores that infect the stem base and spikes throughout the growing season. The infection process is highly dynamic, and it is facilitated by DON. The synthesis of DON (trichothecene) is encoded by *Tri* genes located in four loci. In Poland, the *F. graminearum* population is mainly composed of the 15ADON genotype, and the spread of FHB cannot effectively be managed with fungicides during epidemic years. Dynamic gene flows in field populations enable the pathogen to rapidly adapt to environmental changes and overcome wheat resistance to FHB. The emergence of fungicide-resistant *F. graminearum* strains significantly compromises the quality of wheat crops, but the associated mechanisms have not been sufficiently investigated to date. In addition, although some biopreparations are promising and effective in small-scale field trials, very few have been commercialized. Extensive research into pathogen populations, the development of new resistant wheat varieties, and the use of effective fungicides and biopreparations are required to produce wheat grain that is free of mycotoxins.

## 1. Introduction

Durum wheat (*Triticum turgidum* L. ssp. *durum* Desf.) is particularly susceptible to *Fusarium* pathogens because, compared with the major resistance quantitative trait locus (QTL, *Fhb1* gene, which contributes 25 to 41.6% of the resistance to FHB) identified in bread wheat, the effects of the QTL resistant to FHB detected in tetraploid wheat are relatively small (<12%) [1,2]. Next to common wheat, rice, and maize, durum wheat is a major edible grain and a staple food around the world. Durum wheat is cultivated on an estimated 16 million hectares globally, with a total production of 38 million tons per year [3]. Durum wheat is grown on an area of 2 million ha in Turkey and Canada, and more than 1.5 million ha in Algeria, Italy, and India [4]. Research into high-yielding and drought-resistant durum wheat genotypes [5] creates opportunities for introducing this crop into regions where it was not previously grown [4]. At present, durum wheat is cultivated in Northern Europe [6], as well as in Poland, where it occupies a small area of around 2000 ha [7]. Durum wheat grain is mainly processed into semolina for the production of pasta, bread, biscuits, breakfast cereals, groats, dry gluten, and malt [8,9]. The growing popularity of durum wheat around the world can be attributed to the unique characteristics of its grain (high content of carotenoids, protein, and gluten; hard and vitreous endosperm), which are harnessed in the production of pasta with a desirable yellow color and firm texture that are highly appreciated by consumers [6]. Durum wheat cultivated in the Mediterranean Region is the only economically important species of tetraploid wheat. This allotetraploid species (*Triticum turgidum* L., 2n = 4x = 28, AABB genomes) was derived through intergeneric hybridization and polyploidization of two diploid grass species, *T. urartu* (AA genome) and a diploid B genome associated with *Aegilops speltoides* (BB genome) [1].

Durum wheat yields are affected by biotic (pathogens and pests) and abiotic stressors related to environmental conditions and their interactions with genetic traits [10]. Pathogens can decrease global wheat yields by as much as 21.5% [11]. Fusarium head blight (FHB), a ubiquitous disease of wheat caused by more than 20 species and subspecies of the genus *Fusarium*, including *Fusarium graminearum* (teleomorph *Gibberella zeae* Schwabe), poses a particular threat to durum wheat and common wheat [11,12]. *Fusarium graminearum* is the predominant *Fusarium* species in many regions of Europe [13], Asia [11,12], and North America [14]. Unsurprisingly, a survey of the most important plant-pathogenic fungi revealed that *F. graminearum* was one of the top ten pathogens due to its scientific and economic importance [15]. The prevalence of FHB is influenced by environmental factors such as solar radiation, precipitation, temperature, and CO_2_ levels. The characterization of the linkages between FHB epidemics and environmental changes plays a key role in predictive models and protection methods [16]. Fusarium head blight causes significant losses in wheat production because infected grain is shriveled, small, characterized by low mass and low quality, and often contaminated with mycotoxins that are harmful to humans and animals. The main mycotoxins produced by *Fusarium* species include trichothecenes, zearalenone (ZEA), moniliformin (MON), fumonisins, beauvericin, enniatins (ENNs), and fusaproliferin [10].

The aim of this review article was to describe the structure of *F. graminearum* populations, the infection process, mycotoxin production, the presence of mycotoxins in wheat grain, and the emergence of isolates resistant to fungicides in Poland and other Central European countries.

## 2. Spread and Infection Process of *Fusarium graminearum*

In the agricultural environment, *F. graminearum* survives saprotrophically on harvest residues, where ascospores and macroconidia serve as the main disease inocula of FHB. Ascospores (sexual spores) are released into the air by the perithecia and act as the primary inoculum. Perithecia contain numerous tissue types, produced at specific stages of perithecium development. These include (in order of appearance) formation of the perithecium initials (which give rise to the ascogenous hyphae), the outer wall, paraphyses (sterile mycelia that occupy the center of the perithecium until the asci develop), the asci, and the ascospores within the asci [17]. Macroconidia (asexual spores) are produced by phialides in infected plants and can be transported to other plants throughout the growing season [18]. *Fusarium graminearum* is a highly aggressive pathogen that poses the greatest threat to wheat in the flowering stage [19].

**Figure 1:**
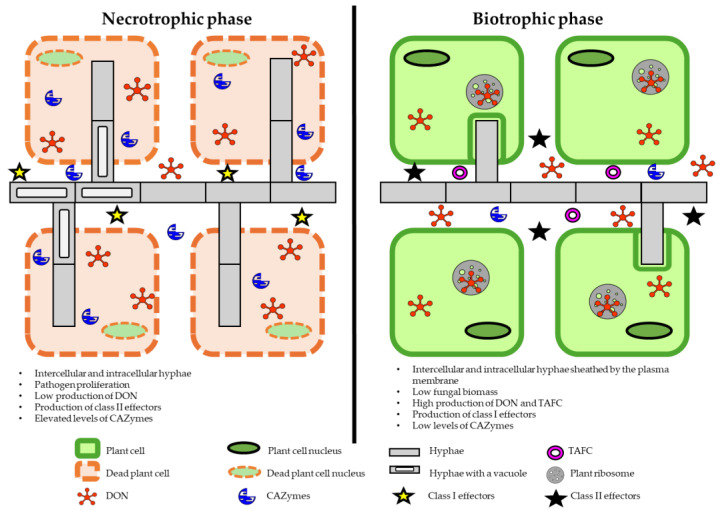
Spatiotemporal model of wheat infection with *Fusarium graminearum.* The model presents the transcriptional regulation of the identified and putative virulence strategies in asymptomatic and symptomatic stages of infection in wheat tissue. Carbohydrate-active enzymes (CAZymes) catalyze the synthesis, degradation, and modification of carbohydrates. DON—deoxynivalenol; TAFC—triacetylfusarinine C. Source: own elaboration based on [25,26].

**Figure 2:**
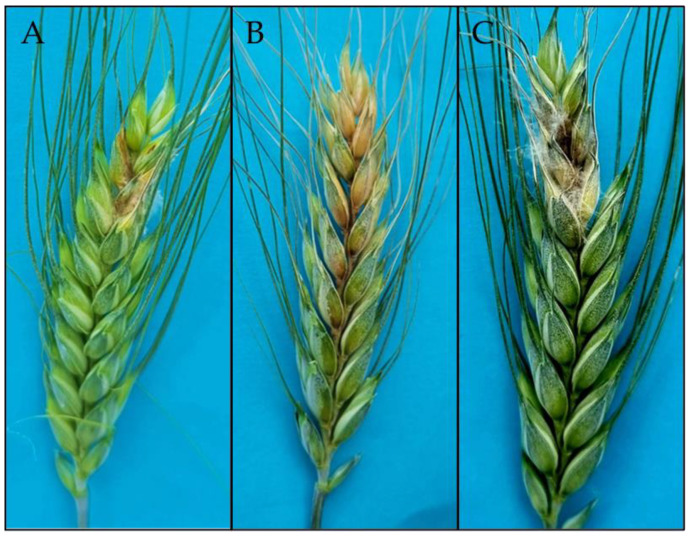
Symptoms of Fusarium head blight on durum wheat cv. Durasol spikes inoculated with the 15ADON genotype of *Fusarium graminearum* after 48 (**A**), 72 (**B**), and 120 (**C**) hours after inoculation.

During the infection process, the pathogen produces secondary metabolites, DON and 15ADON, whose concentrations peak in wheat spikes three days after anthesis [20]. Wheat theca are most sensitive to infection, and they are colonized by fungal hyphae that penetrate top and bottom spikelets [20,21]. *Fusarium graminearum* also infects other parts of wheat spikes, including glumes, lemmas, and palea [21]. This hemibiotrophic pathogen colonizes host tissues as a biotrophic fungus, produces DON, and destroys host cells in the necrotrophic phase. The biotrophic (asymptomatic) phase lasts 24–84 h (Figure 1) [22]. *Fusarium graminearum* spores germinate 5–12 h after inoculation, and germ tubes are usually short and do not target specific topographical features in host plants [23,24,25]. In the biotrophic phase, hyphae grow on the surface of plant tissues or penetrate intercellular spaces, but plant cells remain viable [25]. In a study by Qiu et al. [25], invasive hyphae with a diameter of 1 µm penetrated wheat tissues 16 h after inoculation with *F. graminearum*. In infected cells, thick hyphae changed into narrow hyphae in the cell well and penetrated the neighboring cells. Interestingly, the first infected wheat cells remained viable because the hyphae of the pathogen were still sheathed by the host plasma membrane [25]. *Fusarium graminearum* produced large quantities of deoxynivalenol (DON), triacetylfusarinine C (TAFC), and class 2 effectors in the biotrophic phase, and class 1 effectors in the necrotrophic phase [26]. The first symptoms of the necrotrophic phase in the form of brown spots on glumes were observed after 24–84 h (Figure 2). Hyphal networks and numerous invasive structures were noted on plant surfaces two days after inoculation [23]. Infected spikes were bleached and necrotic, and the peduncle turned black. Symptoms of infection spread from the inoculated apical spikelets to the bottom of the spike. White fungal hyphae were observed on some wheat spikes [22,23] (Figure 2).

Most of the *F. graminearum* life cycle is linked with the host plant. This pathogen belongs to the phylum *Ascomycota*, which is characterized by a long binucleate phase. During this phase, two genetically separate nuclei are coupled inside cells. *Fusarium graminearum* is also homothallic, which implies that it is capable of producing numerous sexual spores (ascospores) without the involvement of a sexually unique partner. The meiotic cycle of *F. graminearum* is controlled by mating-type loci (MAT) MAT1-1 and MAT1-2 [27].

## 3. *Fusarium graminearum* Species Complex, Genotypes/Chemotypes, and Populations

Due to considerable genetic variability in *F. graminearum* populations, this pathogenic species is often referred to as the *F. graminearum* species complex (FGSC). The five most widely researched FGSC species are *F. asiaticum* (47 studies), *F. meridionale* (36), *F. boothii* (20), *F. cortaderiae* (18), and *F. austroamericanum* (9), whereas other species have been identified sporadically [28]. The morphological differences between FGSC species do not exist or are too subtle to support accurate identification [29,30,31].

*Fusarium graminearum* can produce five types of type B trichothecenes, of which three are known to cause gastrointestinal disorders, skin irritation, and neuroendocrine changes [32,33,34]. Selected strains produce DON and its acetylated derivatives 3-acetyl-DON (3ADON genotype) and 15-acetyl-DON (15ADON genotype), as well as nivalenol (NIV) and its acetylated derivative 4-acetyl-NIV (NIV/4ANIV genotype). The 15ADON genotype of *F. graminearum* is most frequently identified in Canada [14], China [11,12], and the USA [35]. In Poland, research into *F. graminearum* genotypes revealed an increase in the number of isolates belonging to the 15ADON genotype [13,36]. In 2009, the *F. graminearum* strains isolated from wheat grain by Wiśniewska et al. [36] belonged exclusively to the 3ADON genotype, whereas in 2019, Duba et al. [37] classified only 46.2% (n = 6) of the isolates to the 3ADON genotypes and the remaining isolates (n = 7) to the 15ADON genotype. In 2016–2017, Bilska et al. [13] confirmed the predominance of the 15ADON genotype but did not detect the NIV genotype in Polish populations of *F. graminearum* (n = 31). In the work of Kulik et al. [38], 76% (n = 25) of *F. graminearum* strains were identified as 15ADON, 8% (n = 6) as 3ADON, and only 6% (n = 2) as NIV genotypes. All strains identified in Western Europe have been classified as 15ADON [38]. In Germany, the 15ADON genotype was also predominant in infected wheat spikes, whereas the prevalence of the 3ADON genotype was lower (6.8%, n = 338), and the prevalence of the NIV genotype was lowest (1.2%) [39]. These observations give cause for concern because the 15ADON genotype is much more virulent than NIV [40]. In contrast, Amarasinghe et al. [41] reported that the prevalence of FHB, the percentage of Fusarium-damaged kernels (FDKs), and the DON content of grain were always higher in wheat cultivars inoculated with 3ADON than 15ADON genotypes of *F. graminearum*. Novel type B trichothecenes, NX-2 and NX-3 (NX), have recently been discovered in *F. graminearum*, but they have not been identified in Poland to date [38]. In the USA, 2.8% of strains in a collection of 463 *F. graminearum* isolates were classified as NX-2 [42]. NX trichothecenes play an important role in the initial stage of *F. graminearum* infection and the spread of FHB [43].

According to research, *F. graminearum* populations are highly diverse and evolve independently in distinct regions [35,38,42]. Kulik et al. [38] identified two major populations of *F. graminearum* in Europe: East European (EE) and West European (WE). The WE population consisted of 28 strains from France, Germany, Luxembourg, and the Netherlands, whereas the EE population included 32 strains from Eastern Europe, mostly Poland and Russia. Two separate populations of *F. graminearum*—endemic (NA1/15ADON) and introduced (NA2/3ADON)—were identified in the USA. In addition, Kelly and Ward [42] assigned a single 3ADON isolate (F328) and the majority (60%) of NX-2 *F. graminearum* to a third genetic population referred to as NA3. The remaining eight NX-2 isolates had admixed genomes, as evidenced by the fact that they shared most of their ancestry with the NA1 population and some ancestry with the NA2 population. In turn, a collection of 213 strains isolated from 13 German fields separated by a distance of 10–500 km was described as a single population with a high degree of sexual recombination [35]. The cited authors argued that high gene flow in field populations enables the pathogen to rapidly adapt to changes in the environment caused by the introduction of resistant cultivars, the use of fungicides, and a warming climate. According to Talas et al. [39], new haplotypes of *F. graminearum* can emerge through sexual recombination between the existing haplotypes in the field (such as maize residues) and haplotypes introduced to the field (e.g., with wheat grain).

## 4. Trichothecene Synthesis—The Key to the Infection Process

**Figure 3:**
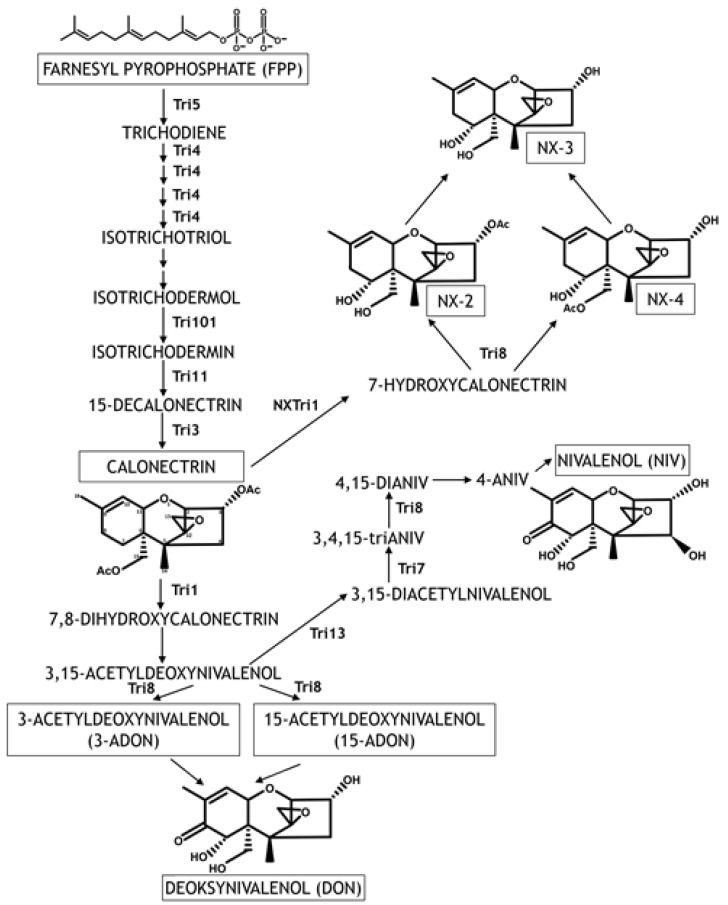
Trichothecene biosynthesis pathway in FGSC strains (modified according to [45,56,57,58]). The atoms in the EPT ring are numbered for calonectrin as the common precursor to TCT-A and TCT-B. The enzymes catalyzing the reactions are described next to the arrows. Arrows without enzymes denote stages with unknown enzymes/genes or reactions that proceed independently.

Trichothecenes (TCT) are the most ubiquitous mycotoxins produced by FGSC. Trichothecenes with a tricyclic 12,13-epoxytrichothec-9-ene (EPT) core structure are secondary metabolites known as sesquiterpenoids (Figure 3). Type A (TCT-A) and type B (TCT-B) trichothecenes are differentiated based on the substitution at the C-8 position. Type A trichothecenes are characterized by a hydroxyl group, an ester, or no substituent at C-8, whereas TCT-B carry a keto group at this position [44]. Type B trichothecenes are most prevalent and include NIV, DON, and their acetylated derivatives (4ANIV, 3ADON, and 15ADON). Type A trichothecenes produced by FGSC strains are NX toxins (NX-2, NX-3, NX-4, NX-5 and NX-6) that do not have an oxygen substitution at C-8, similarly to TCT-A produced by other *Fusarium* species, such as 4,15-diacetoxyscirpenol (DAS). Toxins NX-3 and NX-6 have an identical structure to DON and NIV, respectively, excluding the C-8 substituent. NX-2 and NX-4 (acetylated derivatives of NX-3) and NX-5 (acetylated derivative of NX-6) are characterized by structural similarity to 3ADON, 15ADON, and 4ANIV, respectively. Naturally occurring FGSC strains capable of producing NX-4, NX-5, and NX-6 have not been identified to date [45]. However, NX-4 was identified in vitro in rice cultures [46].

The synthesis of TCT is encoded by *Tri* genes located in four loci. The core TRI cluster consists of 12 genes located on chromosome 2, *Tri1-Tri16* loci on chromosome 1, *Tri101* on chromosome 4, and *Tri15* on chromosome 3 [32,34,41,47]. The core TRI cluster on chromosome 2 contains nine genes encoding enzymes that catalyze DON biosynthesis (*Tri3-Tri5*, *Tri7-Tri9*, *Tri11*, and *Tri13-14*), two genes encoding transcription factors (*Tri6* and *Tri10*), and one gene encoding a protein that participates in the export of mycotoxins (*Tri12*) [32,34,47,48,49]. Transcription factors containing the Cys_2_-His_2_ zinc finger domain are encoded by *Tri6* and *Tri10* genes and regulate the accumulation of *Tri* gene transcripts and other genes with diverse functions. *Tri10* regulates the expression of *Tri6*. Furthermore, the biosynthesis of TCT is regulated by long non-coding RNA (RNA5P) that is transcribed from the *Tri5* promoter region and suppresses the expression of *Tri5* [50].

The biosynthesis of TCT is initiated by the trichodiene synthase enzyme, a virulence factor encoded by the *Tri5* gene that catalyzes the cyclization and isomerization of farnesyl pyrophosphate (FPP) to trichodiene [51]. *Tri5* mutants show symptoms of infection at the site of inoculation, but the infection does not spread to the neighboring spikelets [52]. The following nine reactions leading to the synthesis of calonectrin are catalyzed by the trichodiene oxygenase enzyme encoded by the *Tri4* gene and enzymes encoded by *Tri101*, *Tri11*, and *Tri3* [32,34]. The quadruple oxidation of trichodiene, which precedes non-enzymatic isomerization and cyclization, leads to the formation of isotrichodermol that undergoes acetylation, hydroxylation, and repeated acetylation to form calonectrin. Calonectrin is a common precursor to DON, NIV, their acetylated derivatives (3-ADON, 15-ADON, 4-ANIV), NX toxins, and their ANX derivatives (NX-2 and NX-4) [14]. In NIV-producing *F. graminearum* strains, the biosynthesis pathway is maintained by the product of the *Tri1* gene, which is transformed to 4-ANIV and then to NIV by enzymes encoded by *Tri13*, *Tri7*, and *Tri8*. *Tri7* and *Tri13* genes are not active in *F. graminearum* strains producing DON and its acetylated derivatives, and DON is biosynthesized directly from calonectrin [32,34,41,53]. Calonectrin is transformed into the acetylated derivatives of DON (3ADON and 15ADON) and DON with the involvement of ITD C-15 hydroxylase encoded by the *Tri1* gene and esterase encoded by the *Tri8* gene [32,41]. In FGSC strains producing TCT-A and TCT-B, polymorphisms were identified in the nucleotide sequence of the *Tri1* gene [46,54]. In strains producing TCT-B, the *Tri1* enzyme catalyzes oxidation reactions at positions C-7 and C-8 of the EPT group [55]. In FGSC strains producing NX toxins, the NXTri1 enzyme catalyzes the addition of the hydroxyl group to calonectrin only at position C-7 and can lead to the synthesis of the acetylated derivatives of NX toxins [46].

## 5. The Occurrence of FHB and Mycotoxins in the Field

Central Europe is a region with a temperate climate and average precipitation, but in recent years, wheat plants have been subjected to abiotic stresses caused by prolonged dry spells and considerable temperature fluctuations [7,59]. In 2007–2018, *F. graminearum* was identified on 1–55.1% of common wheat kernels and 2.1–48% of durum wheat kernels in Poland (Table 1). The population size of *F. graminearum* has increased due to weather conditions during wheat flowering or the sensitivity of wheat cultivars/species to infections caused by *Fusarium* spp. The spread of *F. graminearum* can also be attributed to the increase in the area under maize in Poland [13,60,61,62].

Commission Regulation (EU) 2024/1022 of 8 April 2024 amending Regulation (EU) 2023/915 as regards the maximum levels of DON in food decreased the maximum admissible concentration of DON in unprocessed grain to 1000 µg/kg for common wheat and 1500 µg/kg for durum wheat [70]. Research conducted in Poland between 2009 and 2023 demonstrated that all grain samples contained more than one toxin [7,36,59,60,62,64]. Common wheat and durum wheat grain is generally contaminated with DON, MON, and ZEA [7,36,64], but Wachowska et al. [59] identified 11 mycotoxins, including DON, MON, ZEA, several ENNs, NIV, culmorin, and aurofusarin, in durum wheat grain. In Poland, the average content of DON was 30–69,150 µg/kg in common wheat grain and 106–76,700 µg/kg in durum wheat grain (Table 1). In the work of Wiśniewska et al. [36], maximum DON levels were exceeded in all samples. A high concentration of DON in durum wheat grain was also reported by Gorczyca et al. [64], in particular in the Polish cultivar “Komnata” (maximum DON levels were exceeded in 2012/2013 and 2013/2014 seasons). The high content of DON in wheat grain produced in Poland is surprising in the context of the results reported in the Czech Republic, Hungary, and Italy [65,66,69,71]. In most cases, DON concentrations were associated with a higher prevalence of *F. graminearum* in grain [36,60]. However, Góral et al. [61] and Mesterhazy [71] have argued that elevated DON levels can be attributed to varietal resistance to infection and mycotoxin accumulation. Despite the fact that many cultivars are resistant to *Fusarium* infections [71,72], fungicides remain one of the main FHB management strategies in many regions of the world where resistant cultivars are not available. In Poland, fungicides are not sufficiently effective in reducing the symptoms of FHB, protecting grain against *Fusarium* pathogens, and decreasing DON levels in grain. Demethylation inhibitor (DMI) fungicides, in particular tebuconazole, metconazole, and prothioconazole, are most effective in suppressing FHB and decreasing mycotoxin accumulation [7,59,73]. To effectively minimize the spread of FHB, these fungicides should be applied during anthesis. However, according to some reports, the application of triazole and strobilurin fungicides stimulated DON production under field conditions [74]. The associated mechanisms were elucidated in an RT-qPCR analysis by Kulik et al. [75] who observed an increase in the levels of *Tri4*, *Tri5*, and *Tri11* transcripts in the cultures of all *F. graminearum* isolates with the addition of sublethal concentrations of propiconazole. Duan et al. [76] also found that QoIs can stimulate DON production and upregulate the expression of *Tri5* and *Tri6* genes.

## 6. Fungicide Resistance in *Fusarium graminearum* and Wheat Biological Control

The use of fungicides is one of the strategies for managing FHB in wheat, including in integrated crop protection systems, because most wheat cultivars, in particular durum wheat cultivars, are susceptible to this disease [77]. Directive 2009/128/EC on the sustainable use of pesticides was the first legal regulation to comprehensively address the use of plant protection products in Europe. As of January 2014, all EU Member States have to abide by the principles of Integrated Pest Management (IMP) as the main crop protection strategy [78]. Fungicides containing only one active ingredient from the DMI group (prothioconazole, metconazole, tebuconazole, bromuconazole), the quinone outside inhibitor (QoI) group (azoxystrobin), and fungicides containing the above compounds in combination with other triazoles, QoIs (fluoxastrobin, trifloxystrobin), and succinate-dehydrogenase inhibitors (SDHI, bixafen, sedaxane, benzovindiflupyr, fluopyram, and boscalid) are recommended in the European Central Zone [79] (Table 2).

Demethylation inhibitors suppress the demethylation of the 14-C in lanosterol, a precursor to ergosterol that is responsible for the integrity of fungal cell walls. When the production of sterol 14α-demethylase (an enzyme of the cytochrome P450 superfamily) is inhibited, sterol ergosterol precursors and free fatty acids are accumulated in cells, which disrupts normal fungal growth [81]. However, excessive and prolonged use of selective DMI fungicides has decreased the sensitivity of fungal strains or increased the proportion of fungicide-resistant FHB pathogens [35,82]. *Fusarium graminearum* harbors three *CYP51* genes (*CYP51A*, *CYP51B*, and *CYP51C*) which share 61.65% similarity in the amino acid sequence but have different functions. The *CYP51A* gene encodes 14α-demethylase and is responsible for intrinsic sensitivity to DMI fungicides [83]. The *CYP51B* gene encodes an enzyme that is primarily responsible for sterol 14α-demethylation and plays an important role in ascospore formation in *F. graminearum*. However, the *CYP51C* gene does not encode a sterol 14α-demethylase but is required for the full virulence of wheat [83].

The resistance or decreased sensitivity of *F. graminearum* strains to selected triazole fungicides have been well documented in recent years. The efficacy of triazole fungicides decreases significantly after several years of intensive use [84]. Unfortunately, cheap triazole fungicides are the only cost-effective treatments, but FHB infections are practically impossible to eradicate in epidemic years [85]. In *F. graminearum*, resistance to DMI fungicides is conditioned by three main mechanisms: (1) changes in the amino acid sequence of the CYP51 protein, which decrease the affinity for azoles [35,83], (2) overexpression of *CYP51* genes due to changes in the promoter upstream region [86], and (3) overexpression of efflux pumps, including ATP-binding cassette (ABC) transporters and those belonging to the major facilitator superfamily (MSF) of transporters, which can reduce sensitivity by expelling DMI fungicides from fungal cells [87]. In recent years, several *F. graminearum* strains that were resistant or less sensitive to tebuconazole have been identified in wheat fields in Germany, China, Lithuania, and Serbia [35,45,88,89]. In Germany, the EC_50_ values of 197 *F. graminearum* isolates from wheat fields ranged from 5.4 to 62.2 μg mL^−1^. These values had a normal distribution with a mean of 22.2 μg mL^−1^ (Table 3) [35]. The isolates did not contain mutations in the promoter regions of three *CYP51* genes, but three non-synonymous mutations and one synonymous mutation were identified in the *CYP51A* gene, five synonymous point mutations were detected in the *CYP51B* gene, and five non-synonymous mutations and four synonymous mutations were identified in the *CYP51C* gene [35] (Table 3). In *F. graminearum* strains isolated in Lithuania, the EC_50_ values for metconazole were lower than the E_50_ values for prothioconazole and tebuconazole [89]. In Serbia, *F. graminearum* strains were also less sensitive to tebuconazole than metconazole [88]. These observations point to an increase in the EC_50_ values for the inhibition of mycelial growth in Central Europe, particularly in regions where fungicides have been used intensively. The efficacy of fungicides targeting *F. graminearum* clearly decreased in the past 20 years. For example, *F. graminearum* strains isolated from fields in the USA between 1981 and 2014 were significantly more sensitive to metconazole (EC_50_ values for all isolates ranged from 0.0071 to 0.1734 μg/mL, with a mean of 0.0369 μg/mL) and tebuconazole (EC_50_ values ranged from 0.0301 to 1.7339 μg/mL, with a mean of 0.3052 μg/mL) than European strains [81].

Strobilurins (QoIs) inhibit respiration by blocking the cytochrome bc1 enzyme complex (also referred to as complex III). Quinone outside inhibitors are associated with a medium-to-high risk of resistance development in pathogens because point mutations can induce significant changes in the configuration of the target site [80]. In many fungi, resistance to strobilurins is caused by mutations in the *cytb* gene, including at positions G143A, G137R, and F129L. The substitution of phenylalanine with leucine at position 129 (F129L) or the substitution of glycine with arginine at position 137 (G137R) is associated with moderate resistance. In turn, the substitution of glycine with alanine at position 143 (G143A) is associated with stronger resistance [94,95]. Resistance to QoI fungicides has rarely been studied in *F. graminearum*, and the results are often contradictory. According to Dubos et al. [96], *F. graminearum* is naturally resistant to trifloxystrobin and other QoIs. The cited authors analyzed 55 *F. graminearum* strains for resistance to trifloxystrobin. The examined strains belonged to three known chemotypes, and they were isolated between 1969 and 2009 in Belgium, Canada, Germany, Italy, Luxembourg, and the USA. In the tested strains, the maximum inhibition of fungal growth achieved for trifloxystrobin ranged from 14% to 65%, and it was not significantly affected by the country of origin or chemotype. No significant differences in resistance levels were reported between chemotypes and countries, and equally high resistance was noted in strains that had been isolated before strobilurin was placed on the market in 1996. Therefore, the authors concluded that *F. graminearum* is naturally resistant to trifloxystrobin and that resistance to QoI is not caused by using strobilurin in agriculture. In contrast, Duan et al. [76] reported that several strobilurin fungicides were highly effective in inhibiting the mycelial growth of *F. graminearum* strains isolated in China, where the EC_50_ value for azoxystrobin was estimated at 0.966 μg/mL. In a study evaluating the sensitivity of a large collection of *F. graminearum* isolates from two regions of Brazil to azoxystrobin and pyraclostrobin, EC_50_ values were very high (up to 329.7 μg/mL) in several strains, but no point mutations responsible for resistance were detected at any of the target spots in these isolates [91]. The vast majority of the tested isolates were sensitive to the studied fungicides, and pyraclostrobin was more effective than azoxystrobin (the mean EC_50_ was 0.330 and 28.06 μg/mL, respectively). According to Luan et al. [92], *F. graminearum* is not highly sensitive to pyraclostrobin (EC_50_ = 4.175–13.399 μg/mL). The cited authors made the valuable observation that the same isolate can have different EC_50_ values, depending on the culture medium, incubation time, and the addition of other substances.

Research has shown that *F. graminearum* is becoming increasingly resistant to triazoles in Central Europe, which prompts the search for new fungicides to effectively target FHB and reduce the pathogen’s ability to biosynthesize mycotoxins. Cyclobutrifluram, a novel SDHI fungicide developed by Sygenta in 2013 [97] and approved for use in Argentina in 2022 [98], has been recommended for controlling fungal diseases of wheat in China. This compound has not yet received regulatory approval in Central Europe. According to the Fungicide Resistance Action Committee, the risk for resistance evolution to SDHI fungicides is medium to high [80]. Fungal succinate dehydrogenase (SDH) is required for the electron respiratory chain and the tricarboxylic acid cycle. Fungal SDH consists of four subunits, including flavoprotein (SdhA), iron–sulfur protein (SdhB), and two other integral membrane proteins (SdhC and SdhD) that are localized in the mitochondria [93,99]. Shao et al. [99] identified six strains resistant to pydiflumetofen in a collection of 6468 *F. graminearum* isolates from various regions of China. All resistant isolates contained point mutation A78V in FgSdhC1 (FgSdhC1^A78V^). Tong et al. [100] examined the resistance of *F. graminearum* strains and determined the EC_50_ value for sedaxane at 0.365 μg/mL (0.188 to 0.784 μg/mL, depending on sedaxane stereoisomers). In a study analyzing the germination of *F. graminearum* spores, EC_50_ values ranged from 1.28 to 2.41 μg/mL for boscalid, 2.03 to 2.98 μg/mL for benzovindiflupyr, and 0.52 to 0.69 μg/mL for fluopyram (Table 3) [100].

Risoli et al. [101] concluded that biological control agents (BCAs) are highly effective in controlling FHB in wheat, reducing mycotoxin levels, and protecting crops. They also found that BCAs were more effective in studies that had been published more recently, under controlled conditions, and in highly susceptible wheat cultivars. However, BCAs were generally less effective than conventional agrochemicals, especially in their ability to reduce pathogen abundance, FHB symptoms, and mycotoxin levels. *Pythium oligandrum* M1 is the only commercial BCA that has been approved for the management of fungal diseases in Central Europe. Its effectiveness has been analyzed by very few studies [102,103]. When applied to suppress the growth of *F. culmorum* and mycotoxin production during wheat malting, *P. oligandrum* reduced *Fusarium* contamination by 20% and DON content by 17%. The tested BCA did not lead to a deterioration in the quality of wheat malts [102]. In the work of Pellan et al. [103], *P. oligandrum* was also able to settle and colonize the palea or the base of the lemma awn, and it quickly produced a large quantity of characteristic oogonia-containing oospores. This BCA suppressed the development of *F. graminearum* on wheat spikes, decreased DON levels, and inhibited the formation of perithecia. Integrated pest management (IPM) strategies that combine chemical and biological methods are highly recommended, and government subsidies for biological control contribute to their cost-effectiveness in Poland [79].

## 7. Conclusions

*Fusarium graminearum* is a virulent and rapidly spreading hemibiotrophic pathogen of wheat that produces harmful mycotoxins. Dynamic gene flows in field populations enable the pathogen to rapidly adapt to environmental changes, overcome plant resistance, and contribute to the emergence of fungicide resistance. The spread of fungicide-resistant and/or -tolerant *F. graminearum* strains appears to be limited in Central Europe, but it may compromise the quality of wheat crops. The mechanisms responsible for fungicide resistance in *F. graminearum* have not yet been fully elucidated. However, low doses of strobilurin and triazole fungicides have been found to alter metabolic processes in fungal cells by enhancing the release and biosynthesis of mycotoxins, which gives serious cause for concern. These observations warrant further research to improve molecular methods for detecting fungicide-resistant strains and strains with a modified ability to produce mycotoxins and to introduce resistant wheat varieties whose microbiome effectively suppresses the development of *F. graminearum* infections.

## Figures and Tables

**Table 1:** Fungi of the genus *Fusarium* colonizing wheat grain and the mycotoxin content of wheat grain in Poland and several European countries.

Country	Year	Plant Species	*Fusarium* *graminearum*	*Fusarium* *avenaceum*	*Fusarium* *poae*	*Fusarium* *sporotrichioides*	*Fusarium* *tricinctum*	*Fusarium* *culmorum*	Deoxynivalenol	Nivalenol	Zearalenone	Moniliformin	Reference
			Percentage of Colonized Kernels						μg kg^−1^				
Poland	2007	*Triticum* *aestivum*	1	1–8.8	4.0–14.0	1–3.0	0.8–5.8	0	nb	nb	nb	nb	[63]
	2009		6.2–38.5	4.4–71.4	0	0	0.2–4.00	7.2–81.2	2040–76,700	nb	20–680	70–120	[36]
	2009		21.3–55.1	15.8–20.5	0	0	0	5.4–42.9	4010–69,150	nb	4–490	nb	[60]
	2011–2013		1–4.5	2.5–11.0	1.0–8.0	0.3–2.8	1.0–6.0	1.10.0	nb	nb	nb	nb	[62]
	2014		0.7–79.5 *	0–106.7 *	9.4–132.4 *	0–113.3 *	0	0–346.2 *	5.8–444.4	0–19.0	0	0	[61]
	2015		1.5–4.9	0	69–76	0	0	16–26	106–1099	25–453	nb	nb	[7]
	2016–2017		46.07–100	3.03–45.45	9.09	0	0	1.75–13.33	nb	nb	nb	nb	[13]
	2011–2013	*Triticum* *durum*	0	8	2.0	0	2.0	9.0	nb	nb	nb	nb	[62]
	2012		12	24	24	0	12	4	254–1030	<LOD	2.0–27	<LOD	[64]
	2013		48	39	0	5	1	5	10–10,879	<LOD	41–307	9–42	
	2014		43	36	1	13	0	3	424–3988	88–155	2.8–21	40–580	
	2018		2.1–9.0	3	0–24	0	0	4.0–20.1	72–2083	13.9–318.9	0.95–64.4	0	[59]
Italy	2009	*Triticum* *durum*	27	22	0	0	0	13	10.7–597.3	41.0–1648.6	nb	nb	[65]
	2010		69	6	1	1	0	7	32.9–512.3	135.6–965.4	nb	nb	
	2009	*Triticum* *aestivum*	16	37	16	0	0	0	9.7–198.1	122–963.6	nb	nb	
	2010		60	15	3	0	0	4	26.3–261.3	21.1–621.5	nb	nb	
Czech Republic	2015	*Triticum* *aestivum*	38	0	9	0	0	8	20	nb	2	nb	[66]
	2016		77	13	53	0	0	2	148	nb	6	nb	
	2017		21	6	75	32	0	0	75	nb	2	nb	
Lithuania	2013–2014	*Triticum* *aestivum*	nb	nb	nb	nb	nb	nb	nb	nb	nb	nb	[67]
	2005–2007		0	17.6	4.4	4.47	0.6	5.7	nb	nb	nb	nb	[68]
	2011–2013		19.1	18.7	4.9	19.1	1	19.4	nb	nb	nb	nb	
Germany	2006–2007	*Triticum* *aestivum*	64.9	0	3	0	3	26.1	nb	nb	nb	nb	[39]
Hungary	2015	*Triticum* *aestivum*	nb	nb	nb	nb	nb	nb	230–1880	nb	50–98	nb	[69]

*—pg 100^−1^ wheat DNA.

**Table 2:** Active ingredients in fungicides targeting FHB that have been approved for use in Central Europe.

MOA	Group Name	Chemical or Biological Group	Active Ingredient	Target Site and Code	Number of Fungicides
*C. respiration*	QoI-fungicides, (quinone outside inhibitors)	Methoxy-acrylates	azoxystrobin	C3 complex III: cytochrome bc1 (ubiquinol oxidase) at Qo site (cyt b gene)	****
			fluoxastrobin		*
		Oxime acetates	trifloxystrobin		*
	SDHI-fungicides (succinate-dehydrogenase inhibitors)	Pyrazole-4-carboxamides	bixafen	C2 complex II: succinate-dehydrogenase	*
			sedaxane		*
			benzovindiflupyr		*
		Pyridinyl-ethyl-benzamides	fluopyram		*
		Pyridine-carboxamides	boscalid		*
*G: sterol* biosynthesis in membranes	DMI-fungicides (demethylation inhibitors)	Triazolinthiones	prothioconazole	G1 C14-demethylase in sterol biosynthesis (erg11/cyp51)	****
		Triazoles	metconazole		***
	(SBI: Class I)		tebuconazole		***

			bromuconazole		**
Microbial			*Pythium oligandrum*		*

According to: [79,80]. ****—very large number of fungicides, ***—large number of fungicides, **—small number of fungicides, *—very small number of fungicides or microbial product.

**Table 3:** Fungicide sensitivity in *Fusarium graminearum* isolates obtained from wheat.

Fungicide	Country of Origin	Year of Isolation	Number of Isolates	Mean EC_50_ (μg/mL)	EC min (μg/mL)	EC max (μg/mL)	Reference
**Triazoles**							
Propiconazole	Germany	2008	197	22.2	5.4	62.2	[35]
Tebuconazole	Brazil	2011–2020	22	0.57	0.06	3.15	[90]
			11	0.05	0.00	0.19	
Metconazole	Lithuania	2021–2022	13	0.789	0.010	2.967	[89]
Tebuconazole				10.877	1.197	25.623	
Prothioconazole				8.751	2.201	22.909	
Tebuconazole	Serbia		13	1.282	0.910	2.570	[88]
Metconazole		2009		0.479	0.090	1.660	
Carbendazim				0.687	0.390	1.410	
**Strobilurin**							
Azoxystrobin	China	2017	32	0.966	0.274	1.240	[76]
Fluoxastrobin				0.841	0.268	1.775	
Azoxystrobin	Brazil	2007–2020	225	28.06	0.260	329.7	[91]
Pyraclostrobin				0.330	0.030	1.130	
Pyraclostrobin	China		nd *	7.083	4.185	13.399	[92]
**SDHI**							
Boscalid	China	2008–2020	6	2.265	1.280	2.410	[93]
Benzovindiflupyr				2.568	2.030	2.980	
Fluopyram				0.597	0.520	0.690	

nd *—no date.

## Data Availability

The data presented in this study are available on request from the corresponding author. The data are not publicly available due to privacy concerns.
